# Innovative Immunization Strategies for Antivenom Development

**DOI:** 10.3390/toxins10110452

**Published:** 2018-11-02

**Authors:** Erick Bermúdez-Méndez, Albert Fuglsang-Madsen, Sofie Føns, Bruno Lomonte, José María Gutiérrez, Andreas Hougaard Laustsen

**Affiliations:** 1 Facultad de Farmacia, Universidad de Costa Rica, San José 11501-2060, Costa Rica; erick.bermudez_m@ucr.ac.cr; 2 Department of Biotechnology and Biomedicine, Technical University of Denmark, DK-2800 Kongens Lyngby, Denmark; albertfuglsang@outlook.com (A.F.M); sofie.foens@gmail.com (S.F.); 3 Department of Biology, University of Copenhagen, DK-2200 København N, Denmark; 4 Instituto Clodomiro Picado, Facultad de Microbiología, Universidad de Costa Rica, San José 11501-2060, Costa Rica; bruno.lomonte@ucr.ac.cr (B.L.); jose.gutierrez@ucr.ac.cr (J.M.G.)

**Keywords:** animal envenoming, antivenom development, immunization, synthetic epitope, recombinant toxin, DNA immunization, neutralization, omics technologies, bioinformatics, high-density peptide microarray technology, snakebite envenoming, scorpion envenoming, spider envenoming

## Abstract

Snakes, scorpions, and spiders are venomous animals that pose a threat to human health, and severe envenomings from the bites or stings of these animals must be treated with antivenom. Current antivenoms are based on plasma-derived immunoglobulins or immunoglobulin fragments from hyper-immunized animals. Although these medicines have been life-saving for more than 120 years, opportunities to improve envenoming therapy exist. In the later decades, new biotechnological tools have been applied with the aim of improving the efficacy, safety, and affordability of antivenoms. Within the avenues explored, novel immunization strategies using synthetic peptide epitopes, recombinant toxins (or toxoids), or DNA strings as immunogens have demonstrated potential for generating antivenoms with high therapeutic antibody titers and broad neutralizing capacity. Furthermore, these approaches circumvent the need for venom in the production process of antivenoms, thereby limiting some of the complications associated with animal captivity and venom collection. Finally, an important benefit of innovative immunization approaches is that they are often compatible with existing antivenom manufacturing setups. In this review, we compile all reported studies examining venom-independent innovative immunization strategies for antivenom development. In addition, a brief description of toxin families of medical relevance found in snake, scorpion, and spider venoms is presented, as well as how biochemical, bioinformatic, and omics tools could aid the development of next-generation antivenoms.

## 1. Introduction

Snakes, scorpions, and spiders belong to a diverse group of venomous animals capable of causing severe envenomings through their bite or sting, which are considered a serious public health issue in many parts of the world [1,2,3]. Of these, snakebite envenoming is considered as having the highest impact, causing approximately 1.8–2.7 million cases annually, leading to 81,000–138,000 deaths [4,5,6]. Furthermore, it has been estimated that scorpion stings are responsible for approximately 1.2 million envenomings each year, of which more than 3250 people die [7], whereas no reliable epidemiological data for spider bites is available in the literature.

Since the first reports on antivenom development in 1894 [8], parenteral administration of plasma-derived antivenoms of animal origin has been the only specific therapeutic option for the treatment of envenomings by animal bites and stings [3,9]. At present, several public and private laboratories around the world manufacture such antivenoms. However, in some countries, these products are not readily available or are unaffordable for the victims [10,11,12]. Limited availability and affordability issues are, in part, a consequence of the laborious and costly procedure of the traditional antivenom manufacturing process, which requires keeping venomous animals in captivity and milking them to obtain their venoms [13,14,15]. Venom procurement becomes further complicated in cases where animals deliver low amounts of venom or when the animals do not thrive in captivity [13,16].

Even though antivenoms have generally proven effective and have saved thousands of lives worldwide, various intrinsic features from either the manufacturing process or the nature of the antivenom may restrict their clinical efficacy. Antivenom production involves the repeated immunization (for months to years) of large mammals (mostly horses and sheep), followed by purification of the immunoglobulin G (IgG) antibodies from the hyper-immunized plasma. IgG purification is typically carried out by salting-out procedures using either caprylic acid or ammonium sulfate [9,14,17,18,19]. Additionally, most manufacturers produce F(ab’)_2_ antivenoms by introducing a pepsin digestion step [20]. As a result, antivenoms consist of a mixture of both toxin-neutralizing antibodies and antibodies against all other antigens that the immunized animal has come across throughout its life, resulting in a low titer of therapeutically relevant antibodies [21,22]. CroFab [23], an antivenom purified by affinity chromatography using snake venom coupled to a matrix, is the only exception. However, affinity purification greatly increases production costs. Moreover, the toxicity of some venoms largely depends on poorly immunogenic low molecular mass toxins. This discrepancy between toxicity and immunogenicity generates an unbalanced antibody content in antivenoms, with a major fraction of the antibodies targeting immunogenic, but non-toxic, high molecular mass components, and a minor fraction of the antibodies targeting highly toxic, but poorly immunogenic components [24,25,26,27,28].

In order to reduce the public health burden caused by bites or stings of venomous animals, current biotechnological tools have been applied to improve antivenom production (reviewed in [29,30,31]). One useful approach has focused on antivenom production independent of venom use. Here, several modern immunization techniques have been explored, such as the utilization of synthetic peptide epitopes, recombinant toxins (or toxoids), consensus toxins, and DNA strings. Apart from leaving aside venom use and, consequently, the need to keep venomous animals in captivity, antivenom production based on new immunization approaches is considered quite feasible, since it can readily be adapted to current manufacturing platforms without introducing significant modifications. In addition, new immunization approaches may offer the possibility of exclusively using clinically relevant toxins for the immunization procedure, likely leading to a higher titer of therapeutically relevant antibodies and an increased antivenom neutralizing capacity. Such higher titers might, in turn, possibly result in safer products (with lower propensity to cause adverse reactions), as the total dosage of antibodies (which are heterologous proteins) required for venom neutralization would be lower. Moreover, since antivenoms obtained using novel immunization strategies do not differ in nature from traditional antivenoms, regulatory approval processes should not restrain their introduction into the market. However, despite these advantages, inherent batch-to-batch variation is still expected, as a consequence of the dependence on the immune system of immunized production animals [32].

In this paper, we present a comprehensive overview of all studies testing innovative venom-independent immunization strategies for antivenom development. Some examples of alternative venom-dependent approaches are also included. In addition, a summary of the clinically most important toxin families in snake, scorpion, and spider venoms is presented, as well as how complementary biochemical, bioinformatic, and omics tools can be exploited to design cutting-edge immunization protocols. Novel approaches in the therapy of envenomings, such as the development of toxin inhibitors based on aptamers, small molecules, or recombinant antibodies (or fragments thereof) of human or camelid origin are beyond the scope of this review, and can be found elsewhere [32,33,34,35,36,37].

## 2. Clinically Important Toxin Families

Envenoming by snakes, scorpions, and spiders can result in a wide spectrum of pharmacological effects, ranging from localized tissue damage to systemic toxicities. Characteristic toxic effects, which vary depending on the particular venom, include edema, dermonecrosis, myonecrosis, hemolysis, hemorrhage, alterations in coagulation and platelet function, nephrotoxicity, cytotoxicity, and neurotoxicity [6,38,39]. These diverse pathological manifestations are a consequence of the complex composition of venoms, constituted by a mixture of enzymatic and non-enzymatic peptidic and proteinaceous toxins, as well as low molecular mass organic and inorganic components [38,40,41].

Not all venom components contribute to the overall venom toxicity. The clinically most relevant toxins (in terms of lethality) can be determined with the Toxicity Score, a parameter that takes into account both potency (median lethal dose, LD_50_) and abundance of the individual toxins [27,42]. Studies on the efficacy of antivenom in neutralizing key venom toxins have underscored a discrepancy between toxicity and immunogenicity [24,27,43,44]. Many immunogenic venom components have been shown to be irrelevant for the overall toxicity, while some highly toxic venom components are poorly immunogenic, or even immunosuppressive [45], with existing antivenoms sometimes being unable to effectively neutralize them [24,27,44]. Generally, immunogenicity correlates with the molecular size of the toxin, with toxins of low molecular mass being less immunogenic compared to high molecular mass components [24,27,43,44]. Knowledge on venom composition, toxicity of particular components, and toxin immunogenicity is thus imperative for antivenom development. Hence, the identification of the most relevant toxins in venoms of high medical impact is of paramount relevance for the design of optimal mixtures of toxins for immunization. In the following, a brief summary of the clinically most important snake, scorpion, and spider toxin families is presented.

### 2.1. Snake Venom Toxin Families

**Figure 1:**
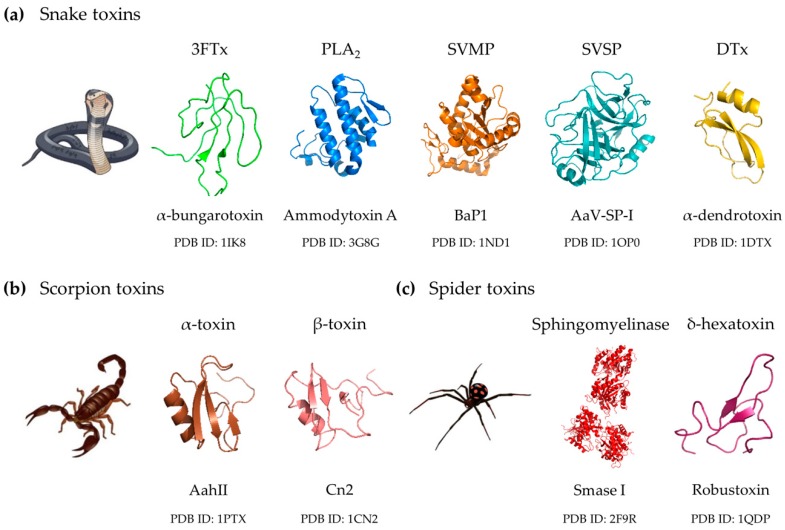
3D structures of representative toxins belonging to each clinically relevant toxin family. (**a**) Snake toxins. (**b**) Scorpion toxins. (**c**) Spider toxins. 3FTx, three-finger toxin; PLA_2_, phospholipase A_2_; SVMP, snake venom metalloproteinase; SVSP, snake venom serine proteinase; DTx, dendrotoxin; BaP1, *Bothrops asper* P-I-type metalloproteinase; AaV-SP-I, *Agkistrodon actus* serine proteinase I; AahII, *Androctonus australis* hector toxin II; Cn2, *Centruroides noxius* Hoffmann toxin 2; Smase I, *Loxosceles laeta* sphingomyelinase I; PDB ID, Protein Data Bank accession ID. Images were created using PyMOL (The PyMOL Molecular Graphics System, Version 2.2 Schrödinger, LLC).

Despite the complexity of snake venoms, most of the medically relevant snake toxins have been found mainly to belong to a handful of toxin families (Figure 1a) [46,47,48]. The clinically most important toxin families include (i) three-finger toxins (3FTxs), (ii) phospholipases A_2_ (PLA_2_s), (iii) snake venom metalloproteinases (SVMPs), and (iv) snake venom serine proteinases (SVSPs) [46]. Additionally, (v) dendrotoxins will also briefly be presented due to their importance for the notorious mamba species [49].

#### 2.1.1. Three-Finger Toxins

3FTxs are found in the venoms of elapids (including sea snakes and terrestrial elapids), ‘colubrids’ (*sensu lato*), and few viperids [46]. These toxins are non-enzymatic polypeptides of typically 60–74 amino acid residues with a shared common scaffold of three β-stranded loops extending from a central, hydrophobic core with four conserved disulfide bonds. Members of the family include the neurotoxic α-toxins, κ-toxins, and muscarinic toxins that target muscle nicotinic cholinergic receptors (nAChRs), neuronal nAChRs, and subtypes of muscarinic receptors, respectively [46]. Another class of 3FTxs are the fasciculins, which inhibit the enzyme acetylcholinesterase, thus preventing acetylcholine from being broken down, leading to its accumulation at the synapse. This interference of neuromuscular inhibition induces fasciculations in muscles [46]. In addition, some elapid venoms contain 3FTxs, which exert cytotoxic activity, hence causing tissue necrosis [50].

#### 2.1.2. Phospholipases A_2_

PLA_2_s are found in the venoms of Viperidae, Elapidae, and Colubridae (*sensu lato*) snakes [6]. The lengths of PLA_2_s vary from 119–134 amino acids, and they share a common scaffold of four main helices with seven intrachain disulfide bonds [51]. PLA_2_s exert a wide variety of toxic activities, including myotoxicity and neurotoxicity, either dependent or independent of their catalytic activity [51,52]. The toxicity is caused by an initial plasma membrane perturbation, which promotes a large increase in the cytosolic Ca^2+^ concentration that leads to degenerative events, including impairment of mitochondrial function. Some PLA_2_s target presynaptic nerve terminals in the peripheral nervous system causing paralysis, whereas others target both the nervous and muscular systems, or act on skeletal muscles. The latter cause irreversible muscle damage, which can induce systemic myotoxicity, e.g., rhabdomyolysis with myoglobinuria, leading to acute kidney injury [51]. Venom PLA_2_s also exert other activities, including inhibition of coagulation, intravascular hemolysis, and are strongly pro-inflammatory [52].

#### 2.1.3. Snake Venom Metalloproteinases

Zinc-dependent SVMPs are a major component of venoms in the snake family Viperidae, but are also present in species of Colubridae (*sensu lato*) and Elapidae [53,54]. SVMPs can be divided into three classes (P-I, P-II, and P-III), depending on their domain composition, with molecular masses of 20–30 kDa, 30–60 kDa, and 60–100 kDa, respectively [54]. SVMPs induce local and systemic hemorrhage by hydrolyzing components of the extracellular matrix, including collagen IV in the basement membrane of capillary blood vessels [6,53]. Their cleavage of cell-cell junctions also promotes the mechanical weakening of the microvessel wall, leading to extravasation [6]. In addition, SVMPs cause myonecrosis, blistering, dermonecrosis, edema, and coagulopathies, and like PLA_2_s, induce pain and inflammation [53].

#### 2.1.4. Snake Venom Serine Proteinases

SVSPs are found in venoms of the snake families Viperidae, Elapidae, and Colubridae (*sensu lato*) [55]. The molecular masses of SVSPs vary between 26 and 67 kDa, depending on the extent of glycosylation. SVSPs are trypsin-like enzymes, but with variable macromolecular substrate specificity, acting by a common catalytic mechanism that includes a reactive serine residue [55]. SVSPs interfere with blood coagulation, fibrinolysis, blood pressure, and platelet aggregation. SVMPs and SVSPs act in the coagulation cascade, which can promote intravascular coagulation, but more often lead to consumption coagulopathy [6]. The resulting incoagulability can contribute to systemic bleeding, especially in venoms that simultaneously contain hemorrhagic toxins [6,55].

#### 2.1.5. Dendrotoxins

Dendrotoxins are a family of toxins found among the *Dendroaspis* (mamba) snakes of the family Elapidae. They consist of 57–60 amino acid residues crosslinked by three disulfide bonds, and show structural homology to Kunitz-type serine protease inhibitors, albeit with different pharmacological effects. Dendrotoxins exert their effects by blocking specific subtypes of voltage-dependent potassium channels (Kv1 subfamily in neurons) that facilitate acetylcholine release at peripheral synapses, resulting in an excitatory effect [49,56].

#### 2.1.6. Minor Snake Venom Toxin Families

The toxin families discussed above represent the most important toxins in snake venoms from the pathological and pathophysiological standpoint. However, snake venoms contain various other proteins with lower contribution to venom toxicity. These minor snake venom protein families include C-type lectin-like proteins, cysteine-rich secretory proteins (CRISPs), l-amino acid oxidases, low molecular mass myotoxins (e.g., crotamine), vasoactive peptides, disintegrins, hyaluronidases, natriuretic peptides, and sarafotoxins, among others, which are outside the scope of this paper and have been described elsewhere [6,57].

### 2.2. Scorpion Venom Toxins

The clinically most relevant toxins in scorpion venoms are the scorpion α and β-toxins, both of which are composed of 61–76 amino acid residues cross-linked by four disulfide bonds (Figure 1b). These neurotoxins typically adopt a highly conserved three-dimensional structure comprising an α-helix and three or four-stranded anti-parallel β-sheets with high chemical and thermal stability. These toxins interact with multiple sites on voltage-gated sodium (Na_V_) channels [39,58], reflecting their distinct pharmacological effects and mechanisms of action. The α-toxins (also known as Old World scorpion toxins) target neurotoxin binding site 3, which is localized on the extracellular surface of the Na_V_ channels and impede fast inactivation [58]. Consequently, this leads to prolonged depolarization and excessive neuronal activity. The resulting sympathetic excitation and the endogenous release of catecholamines can cause severe systemic effects, including myocardial injury, pulmonary edema, and cardiogenic shock [39]. In contrast, the β-toxins (also known as New World scorpion toxins) target neurotoxin binding site 4. Electrophysiological studies have revealed that the β-toxins cause a hyperpolarizing shift in the voltage dependence of activation, thus lowering the threshold for action potential firing [58]. Other neurotoxins in scorpion venoms act on voltage-gated potassium and calcium channels. However, these toxins appear to be less important in human envenoming [39]. Severe envenoming is often caused by scorpions of the Buthidae family, nonetheless, in most cases of scorpion stings, only localized pain and minimal systemic involvement follow [39]. Additionally, scorpion venoms contain a range of enzymatic toxins, such as hyaluronidases, metalloproteinases, and phospholipases [33].

### 2.3. Spider Venom Toxins

In the majority of human envenoming cases, spider bites only cause minor effects, although worldwide a few groups of spiders cause more significant effects and are thus medically significant. These groups include (i) the widow spiders (*Latrodectus* spp.), (ii) the recluse spiders (*Loxosceles* spp.), (iii) the Australian funnel-web spiders (*Atrax* spp. and *Hadronyche* spp.), and (iv) the armed or banana spiders (*Phoneutria* spp.) from Brazil [59,60]. In the following, a brief description of representative toxins from each group will be given.

#### 2.3.1. α-Latrotoxin

α-Latrotoxin (α-LTX) is a 130 kDa neurotoxin found in *Latrodectus* venoms that appears to be responsible for the clinical effects in humans resulting in the envenoming syndrome ‘latrodectism’ [60,61]. α-LTX induces neurotransmitter vesicle exocytosis via both Ca^2+^-dependent and independent mechanisms [61]. Latrodectism is characterized by diaphoresis and pain developing gradually and lasting for hours to days. In case of systemic envenoming, non-specific symptoms such as nausea, vomiting, headache, and fatigue are common, but latrodectism is rarely life-threatening [59,60].

#### 2.3.2. Sphingomyelinases D

Sphingomyelinases D (SMases D) are found in *Loxosceles* venoms and are considered the key components involved in the development of dermonecrosis, the main manifestation of the cutaneous form of loxoscelism. SMases D are 31–35 kDa enzymes that hydrolyze sphingomyelin in the outer leaflet of mammalian plasma membranes, resulting in the formation of ceramide (Figure 1c) [62]. Local effects of *Loxosceles* bites vary from small areas of erythema to progression of large areas of ulceration and necrosis that can take months to heal. The often painless bite will in up to 10% of cases result in severe systemic reactions (viscerocutaneous loxoscelism), including shock, complement-mediated intravascular hemolysis, and renal failure [60,62].

#### 2.3.3. δ-Hexatoxins

δ-Hexatoxins (δ-HXTXs) are homologous neurotoxins of about 4.8 kDa, responsible for the severe envenoming symptoms in humans caused by bites of six different funnel-web species (Figure 1c) [63]. Severe systemic envenoming is rare, but the onset of life-threatening effects is rapid. Severe systemic envenoming is characterized by neuromuscular excitation, including paraesthesia and fasciculations, pulmonary edema and massive autonomic stimulation/excitation with generalized diaphoresis, hypersalivation, hyperlacrimation, and hypertension. Without antivenom, further effects like neuromuscular paralysis, coma, hypotension, and multi-organ failure might follow [59]. The δ-HXTXs induce spontaneous repetitive firing and prolongation of action potentials by binding to receptor site 3 on Na_V_ channels, slowing the inactivation of the channels and causing a hyperpolarizing shift of the voltage dependence of activation [61].

#### 2.3.4. Tx2-6

Tx2-6 is a small neurotoxin of about 5.3 kDa found in the venom of *Phoneutria* spiders [64], that like the δ-HXTXs, slows inactivation of Na_V_ channels and causes a shift in the hyperpolarized direction of voltage dependence of activation [65]. A bite from the *Phoneutria nigriventer* spider causes immediate local pain, diaphoresis, piloerection, and erythema. In systemic envenoming cases, non-specific symptoms, such as nausea and vomiting, are observed, as well as autonomic effects, such as tachycardia, hypertension, salivation, and priapism. It can in rare circumstances progress to pulmonary edema [60].

## 3. Innovative Venom-Independent Immunization Strategies

**Figure 2:**
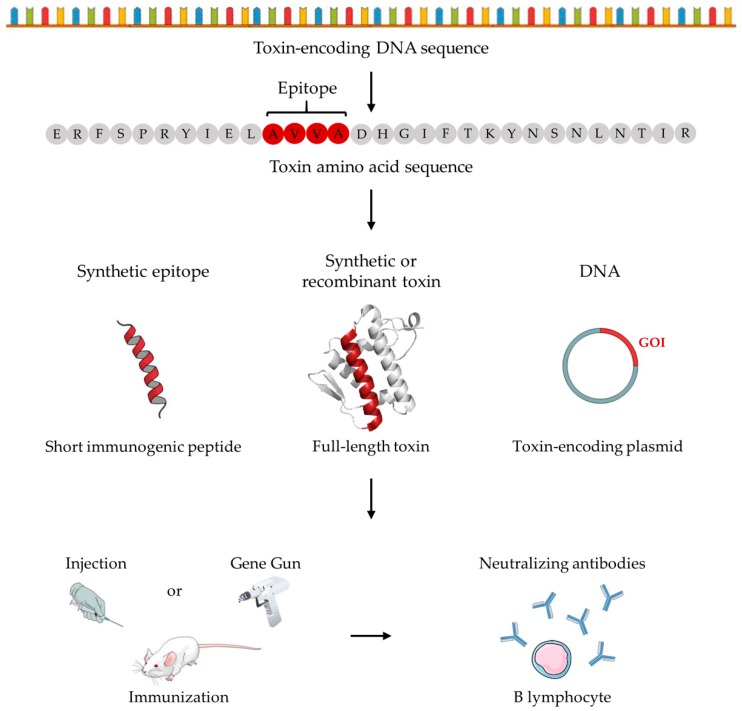
Schematic illustration of the most studied innovative immunization strategies explored for antivenom development. Based on the amino acid sequence of the toxin(s) of interest, short immunogenic peptide(s) or full-length toxin(s), synthetically or recombinantly produced, can be injected into animals. Immunization with the peptide(s) or toxin(s) will lead to a toxin-specific immune response mediated by antibody-producing B lymphocytes. Alternatively, based on the DNA sequence(s) of the toxin(s), a plasmid encoding the gene(s) of interest (GOI) can be transfected into animal cells using a Gene Gun. Following transfection, the animal cells will translate the transcript derived from the plasmid, generating a toxin able to raise a toxin-specific immune response with subsequent production of antibodies by B lymphocytes.

To avoid disadvantages associated with conventional antivenom production, while utilizing existing manufacturing platforms, several researchers have studied the use of recombinant or synthetic toxins and peptides, as well as DNA vaccination strategies to raise therapeutically relevant antibodies via immunization procedures (Figure 2). By removing the current need for venom for antivenom production, and hence for keeping collections of venomous animals, the laborious and potentially dangerous work associated with animal handling can largely be circumvented for personnel [66]. However, venom will still be needed for antivenom quality control and research validation to ensure that novel manufactured antivenoms have adequate efficacy. In the following, an exhaustive compilation of novel venom-independent immunization approaches used in the snake, scorpion, and spider antivenom research fields is provided.

### 3.1. Studies within Snake Antivenom Development

In terms of snake antivenom development using venom-independent immunization techniques, variants of four different approaches have been utilized, summarized in chronological order in Table 1.

The first novel immunization approach concerns the injection of chemically synthesized epitopes of toxins, usually based on epitope mapping studies or epitope prediction using bioinformatic software. The antisera raised using these discovered or predicted epitopes have been reported to achieve both partial and complete neutralization in a range of studies. For instance, myotoxicity in mice induced by *Bothrops asper* myotoxin II (a PLA_2_) was partially inhibited by antiserum raised against a peptide comprising residues 115–129 (coupled to a diphtheria toxoid) [67], while another study found prolongation of survival when mice immunized with three peptides from the C-terminus of ammodytoxin A (a PLA_2_) were challenged with *Crotalus durissus terrificus* and *Vipera ammodytes* snake venom [68]. In another study, complete neutralization of hemotoxicity was reported when rabbits immunized with synthetic mutalysin II epitopes were challenged with *Lachesis muta muta* snake venom [69]. Furthermore, in 1991, Čurin-Šerbec et al. immunized rabbits with four individual synthetic peptides (residues 70–78, 106–113, 113–121, and 125–133) from ammodytoxin A conjugated to keyhole limpet hemocyanin (KLH). Rabbit antiserum targeting peptide residues 106–113 completely abolished lethality when naïve mice were challenged with ammodytoxin A, whereas antiserum against residues 113–121 partially blocked toxicity [70]. In another study by Dolimbek and Atassi in 1996, mice were immunized with three peptides from α-bungarotoxin (a 3FTx from *Bungarus multicinctus* venom), which raised an immune response that was reported to neutralize up to 15 LD_50_s of α-bungarotoxin injected intravenously in the immunized mice [71]. By further conjugating all three peptides to ovalbumin, the protective capacity exceeded that of the mice immunized with native toxin by almost 2-fold. In a separate study from 2016, Cao and colleagues used the bioinformatic software DNAStar and the online IEDB software (http://tools.immuneepitope.org/bcell/) to predict six linear B-cell epitopes of three major toxins from *Deinagkistrodon acutus* snake venom. Murine antiserum raised against a tandem protein of the six linear epitopes was able to protect naïve mice against 4 minimum hemorrhagic doses (MHDs). Here, 1 MHD was defined as the dosage of snake venom required to produce a 10 mm hemorrhagic spot on the visceral side of the back skin, 24 h after intradermal injection [72].

A second novel immunization approach that has been investigated involves the use of full-length synthetic or recombinant toxins as immunogens, and not only toxin epitopes. Compared to epitopes, both in terms of size and structure, it is likely that these proteins serve as better immunogens due to a closer similarity to the native toxins. A native-like structure is crucial for the generation of antibodies able to recognize the native toxins. Furthermore, no prior epitope mapping study is required. On the other hand, there is a risk of the toxins not folding correctly after chemical synthesis or recombinant expression. One study reported complete neutralization of 2 LD_50_s of *Bungarus candidus* snake venom injected intravenously in naïve mice, when venom was pre-incubated with murine antiserum generated by immunization with α and β-neurotoxins (from α and β-bungarotoxin-based RT-PCR and subsequent cloning and protein expression) as immunogens [73]. Furthermore, 3 LD_50_s of a type I α-neurotoxin from *Micrurus diastema* was reported neutralized, when incubated with rabbit antiserum raised against a recombinant α-neurotoxin (called rD.H) and injected intravenously in naïve mice [74]. More recently, the investigation of a short-chain consensus α-neurotoxin was reported by de la Rosa et al. As α-neurotoxins can be difficult to purify from Elapid snake venoms, the sequences of 12 of the most toxic α-neurotoxins from venoms in the Elapidae family were used to generate a recombinant consensus toxin, which was used to immunize rabbits. The consensus toxin was shown to antagonize muscular nAChRs, and the raised immune antiserum was demonstrated to recognize the native toxins from the venom of selected Elapid snakes (particular high titers against *Micrurus elegans*, *M. latifasciatus*, and *M. nigrocinctus* snake venom) [75]. In comparison, the study by Guerrero-Garzón and colleagues reported the generation of protective rabbit antiserum through immunization with a recombinant D.H (rD.H) α-neurotoxin, capable of neutralizing the lethal activity of three *Micrurus* neurotoxins (including rD.H) [74]. Considering the relatively low abundance and poor immunogenicity of 3FTxs in *Micrurus* venoms [43,74,76], supplementing the immunization mixture of *Micrurus* snake venoms with recombinant 3FTxs might enhance the therapeutic efficacy of the generated antivenom.

Thirdly, mimotopes (i.e., molecules mimicking the structure of toxin epitopes, but with non-identical amino acid sequences) have been developed [77,78]. These mimotopes were developed by selecting peptides against immobilized monoclonal antibodies that have shown neutralizing capacity, using *k*-mer peptide libraries and phage display technology. Despite the ability of the immobilized antibodies to recognize different peptide-displaying phages, low sequence similarity existed between the original toxin epitopes and the corresponding mimicked epitope [77,78]. Due to their non-toxic nature, immunizing with epitopes or mimotopes has the advantage and opportunity to allow for increased injection dosage, which may often lead to a more efficient immune response. However, a low molecular mass may decrease immunogenicity, which again could be compensated for, by an increase in injection dosage or by coupling the immunizing peptides with high molecular mass proteins.

Lastly, avoiding chemical synthesis or recombinant expression (with subsequent purification steps), several studies have looked into the opportunity of using DNA immunization. Here, the use of a Gene Gun to inject DNA (bound to gold or tungsten beads) into the animal that is to be immunized has been reported. The injection targets epidermal cells in the abdominal region, which then express and display epitopes for T helper 2 (T_H_2) cells to respond to [79,80,81]. From studies exploring DNA immunization in the snake antivenom field, both antisera with lower [66,79,82] and higher [81,83] neutralization capacities compared to conventional antisera have been reported. Wagstaff and colleagues demonstrated that a mouse antiserum raised using an *Echis ocellatus* SVMP multiepitope DNA string had better neutralization capacity in mice than an antiserum raised in rabbits by whole *E. ocellatus* venom immunization [83]. Azofeifa-Cordero and co-workers reported partial neutralization of hemorrhage induced by the rattlesnake *Crotalus durissus durissus* venom by immunizing with DNA of a P-III type SVMP [84]. In a study by Pergolizzi et al. in 2004, it was demonstrated that immunization by intravenous administration of either a modified α-cobratoxin plasmid alone or delivered by a replication-deficient adenovirus vector conferred protection to mice (100% survival rate), when challenged intravenously with 1 LD_80_ of α-cobratoxin from *Naja kaouthia* [85]. Interestingly, one study reported the combined use of DNA injection followed by immunization with recombinant toxins in mice. Here, 60% survival rate in naïve mice was achieved when mice were challenged intraperitoneally with 3 LD_50_s of *Micrurus corallinus* snake venom pre-incubated with the generated murine antiserum [66].

One of the challenges faced by DNA vaccines/immunization for human use has been insufficient immune responses, despite success in pre-clinical models [86]. Although the antivenom field does not rely on conferring an active vaccination of humans, DNA vaccination has great potential as an immunization technique to obtain exogenous antibodies to be used for envenoming therapy [87]. However, when scaling up from murine models to larger production animals, high IgG titers have failed to be achieved [87]. Some studies have tried to compensate for insufficient immune response in production animals by priming with a DNA immunization and boosting with recombinant proteins [66,88], thus lowering the required amount of protein antigen. DNA priming has also been reported for HIV vaccination [89]. Alternatively, combining DNA immunization with appropriate adjuvants also seems a feasible immunization approach for production of antivenom. A future step in this direction may be inspired by development in other fields, where peptides or antibodies are incorporated in liposomes for targeted payload delivery [90,91,92,93,94]. In this way, the efficacy of the immune response may increase by directly targeting professional antigen-presenting cells (APCs) or T helper cells, instead of having antigen presentation by abdominal epidermal cells targeted by Gene Gun DNA injection.

By relying on DNA sequences rather than synthetically or recombinantly produced peptides or toxins for immunization, less antigen is needed, as each DNA molecule can yield many antigen molecules after translation. It may also prove less toxic for the production animals by lowering peak exposure to toxins, thereby conferring the possibility of utilizing a high DNA immunization dosage, potentially providing a stronger immune response [87]. In the future, this immunization approach might also be further improved by adapting successful approaches from the fields of gene therapy and DNA vaccination research.

### 3.2. Studies within Scorpion Antivenom Development

In the scorpion antivenom field, antivenom producers could benefit from being independent of production animals for venom procurement, as this procedure is quite laborious due to involvement of electrostimulation [16]. As with the snake antivenom field, synthetic epitopes have been developed for immunization research (Table 2, see studies [97,98,99,100,101,102,103]). Several studies found varying degrees of antibody-mediated protection against different antigen dosages. In 1986, Bahraoui et al. immunized rabbits with a synthetic peptide of residues 50–59 of *Androctonus australis hector* toxin II (AahII; a scorpion α-toxin) and used the generated antiserum to protect naïve mice against an intracerebroventricular challenge of 14 LD_50_s of AahII, reporting 80% protection [97]. Interestingly, the study also reported that conjugation of the synthetic peptide to bovine serum albumin (BSA) resulted in antiserum that did not bind the free native toxins, highlighting that the choice of protein carrier should be considered carefully, and that conjugation to carriers might not be a possibility for all immunogens. Chávez-Olórtegui et al. reported partial protection of naïve mice against the toxic fraction of *Tityus serrulatus* venom (TstG_50_), when subcutaneously injecting the pre-incubated toxic fraction with antiserum derived from rabbits immunized with synthetic peptides derived from the non-toxic protein TsNTxP [104]. Calderón-Aranda et al. immunized rabbits with 7 peptides derived from *Centruroides noxius* toxin 2 (Cn2; a scorpion β-toxin) and reported 10–80% protection in naïve mice after these were challenged intraperitoneally with 1 LD_50_ of Cn2 incubated with the rabbit antiserum [98]. Interestingly, the latter study did not observe immunogenicity of all the utilized epitopes, bringing attention to the need for careful epitope selection and validation [98]. Another study reported full neutralization in naïve mice of up to 39.5 LD_50_s of Cn2 injected intraperitoneally after incubating Cn2 with antiserum from rabbits immunized with synthetic epitopes of Cn2, where also discontinuous epitopes were taken into account [99]. By 2002, Alvarenga and colleagues reported the immunization of rabbits with four epitopes from TsNTxP and one epitope from the major lethal component, TsIV, all conjugated to KLH. The rabbit antiserum was able to neutralize 13.5 LD_50_s of TstG_50_-fractionated *T. serrulatus* venom (subcutaneous route) per mL antiserum in naïve mice [100]. Lastly, rabbits were immunized with an 18-residue peptide from a birtoxin-like N-terminus peptide (conjugated to KLH), and the IgGs were purified and reported to neutralize up to 60 LD_99_ of *Parabuthus transvaalicus* scorpion venom (intracerebroventricular route) per mL of IgG [101]. In general, these studies highlight that when selecting epitopes for immunization, focusing on similarity between the most toxic venom components may be highly beneficial. Other than homology, conjugation to KLH seems to be beneficial for increasing the immunization dosages, which may lead to stronger immune responses. This has also been achieved by conjugation of venom components to other protein carriers, such as ovalbumin, BSA, diphtheria toxoid [67], and tetanus toxoid [105]. Lowered toxicity, allowing for increased immunization dosages, can also be achieved by altering the non-epitopic residues, such as it has been done with chimeric toxins, mimotopes, and retro-inverso peptides, as discussed in the next section [98,106,107]. 

Several studies utilizing recombinant or synthetic toxins/peptides for immunization have reported successful results (Table 2, see studies [106,108,109,110,111,112,113,114,115,116,117,118,119,120]). One study found various levels of protection of naïve mice challenged with intraperitoneal or subcutaneous injections of 1–3 LD_50_s of Cn2 from *C. noxius* Hoffmann scorpion venom, when pre-incubated with rabbit antiserum raised against a recombinant Cn5 (a crustacean-specific toxin) [112]. Zenouaki and colleagues reported 66% protection of mice challenged with 5 LD_50_s (intracerebroventricular route) of AahII from *A. australis hector* venom after immunization with a chimeric toxin, where α-aminobutyric acid was utilized to replace cysteines [106]. Mendes et al. reported up to 75% protection against 2 LD_50_s injected subcutaneously in naïve mice, against *T. serrulatus* scorpion venom, when incubating the scorpion venom with antiserum from immunized rabbits prior to injection into the mice. The rabbits were immunized with recombinant Ts1 toxoids (Ts1 is also called TsVIII or Ts-γ) [114]. Protection of 100% was furthermore observed when challenging naïve mice subcutaneously with 14 LD_50_s of native Ts1 after pre-incubation with the rabbit antiserum. In another study, rabbits were immunized with AahI, AahII, and AahIII fused to maltose-binding protein (MBP), yielding antiserum, which neutralized up to 15 LD_50_s/mL (subcutaneous route), when incubating the toxic fraction (Aah-G50) of *A. australis hector* venom with the rabbit antiserum prior to injection into naïve mice. This neutralization capacity was equal to the commercial antivenom available at the time [110]. In the same study, a chimeric toxin consisting of AahI-MBP-AahII was used for immunization of rabbits, but was reported not to bind the native AahI or AahII, indicating that epitopes may lose their immunogenicity if fusion leads to loss of their native conformation, once again highlighting the importance of native-like structures of immunogens for generating antibodies with optimal neutralization capacity [110]. In 1997, 200 LD_50_s of AahII per mg of monoclonal antibody was neutralized in naïve mice (intracerebroventricular route), when pre-incubated with purified IgGs from mouse hybridoma cells, where the mice had been immunized with a synthetic AahII toxoid (with α-aminobutyric acid replacing cysteines) [108]. Furthermore, another study showed that by immunizing mice with a fusion protein of Bot III and ZZ domains of staphylococcal protein A, the immunized mice were protected against 10 LD_50_s (subcutaneous route) of the most lethal toxin, Bot III, from *Buthus occitanus tunetanus* scorpion venom [111]. A study by Jiménez-Vargas and colleagues in 2017 found that antiserum raised against recombinant fusion proteins of thioredoxin-enterokinase cleavage site-Cn2 (Thio-EK-Cn2), Thio-EK-*C. suffusus* toxin II, and Thio-EK-*C. limpidus* toxin I or II could neutralize 3 LD_50_s of *C. noxius*, *C. limpidus*, *C. suffusus*, *C. tecomanus*, *C. elegans*, and *C. sculpturatus* scorpion venom in naïve mice, when pre-incubated with just 0.25 mL of rabbit antiserum and injected intraperitoneally [118]. Lastly, a US patent was granted for the use of 71 gene sequences encoding various scorpion genes for potential DNA immunization, immunization with the corresponding recombinant proteins, or immunoglobulin purification purposes [113]. It is worth mentioning that DNA immunization has yet to be attempted in the scorpion antivenom field.

From the above, it becomes evident that selecting which immunization strategy to use when applying synthetic or recombinant peptides or toxins as immunogens for development of antiserum can be difficult, as results are influenced by several factors. Neutralization capacity depends on the specific IgG titer of the antiserum, which in turn depends on the quantity and immunogenicity of the antigen used for immunization. In addition, the affinity of the antibodies towards the epitopes definitely play a key role. Secondly, the chosen animal model and its intrinsic genetic variance should also be considered. Thus, after optimizing the protocols in small animal species, it is relevant to upscale the protocols to large animals used to manufacture antivenoms. Another factor that makes comparison of antisera particularly challenging is the use of different antiserum volumes and lethal toxin dosages. Standardization in how the protective assays are executed would greatly improve comparability between different methods.

### 3.3. Studies within Spider Antivenom Development

Production of spider antivenom stands to greatly benefit from innovative immunization approaches, as sufficient amounts of venom are hard to procure due to animal maintenance and laborious venom extraction by electrostimulation [123,124,125]. In this antivenom field, synthetic toxins [126], recombinant toxins [124,125,126,127,128,129,130,131,132,133,134,135], synthetic epitopes [107,136,137], and a synthetic mimotope [138] have been used as venom-independent immunization techniques for spider antivenom development. An overview of the different studies is presented in Table 3.

Several studies have reported partial neutralization when utilizing synthetic or recombinant toxins for immunization. Successfully raised antibody responses have yielded antisera, which were tested against venoms or native toxins at different dosages of venom/toxin. For instance, Felicori et al. produced the recombinant dermonecrotic protein I (rLiD1) from *Loxosceles intermedia* and utilized it as an immunogen in mice. A protection of 75% was reported when the immunized mice were later challenged subcutaneously with 2.5 LD_50_s of rLiD1 [129]. Conversely, Magalhães et al. reported a 65% reduction in inflammation and almost complete abolishment of dermonecrosis in naïve rabbits after incubation of *Loxosceles gaucho* spider venom with rabbit antiserum, raised against a recombinant phospholipase D (LgRec1), natively from *L. gaucho* spider venom [124]. More studies report full neutralization of *Loxosceles* sp. venom [127,128,134,139], of which one study reported the development of a more effective antiserum than an existing commercial antivenom on the spider antivenom market. In this study, the antiserum was raised against a sphingomyelinase D in horses and tested in rabbits challenged with *L. intermedia*, *L. laeta,* and *L. gaucho* spider venom, demonstrating neutralization capacity [130].

In 2009, Comis and colleagues reported the use of a synthetic robustoxin from *Atrax robustus* venom, which had been conjugated to KLH and chemically deactivated by preventing disulfide bridge formation, as an immunogen in *Macaca fascicularis* monkeys. After 15 weeks, the immunized monkeys survived a subsequent challenge of 50 µg/kg of *A. robustus* spider venom [126]. Furthermore, Oliveira et al. evaluated the utilization of transgenic mice (engineered to have human MHC class II) for immunization purposes, where the collected murine antisera raised against recombinant sphingomyelinase D (rLid1) were able to inhibit 87% of edema and to neutralize dermonecrosis and hemotoxicity in naïve rabbits [135].

In studies that utilized synthetic epitopes or mimotopes for immunization, mostly the immunized animals were challenged with venom or native toxins [107,125,137,138]. In one of these studies, Fischer and colleagues reversed the primary sequence of robustoxin from *A. robustus* venom and inverted all the chiral centers, yielding a proteolysis-resistant peptide that could be administered orally or intraperitoneally. This method was shown to generate an immune response that saved 7 out of 8 immunized mice [107]. Interestingly, in 2011, de Moura et al. observed that the epitope of LiD1 recognized by a previously reported monoclonal antibody could not be identified by a SPOT epitope-scan. By screening a peptide library via phage display, de Moura et al. found a mimotope mimicking a discontinuous epitope [138]. The mimotope was recognized by the monoclonal antibody and utilized for immunization by liposomal delivery (cholesterol and phosphatidylcholine), which generated an immune response in rabbits. The immunized rabbits were subsequently challenged with 10 µg of *L. intermedia* spider venom. Here, 60% dermonecrotic protection, 80% protection against hemorrhage, and higher protection against edema than rabbits immunized with the *L. intermedia* spider venom was reported [138]. More studies have utilized discontinuous epitopes as well [77,99,126,131,140].

Three studies by Mendes, Figueiredo, Lima, and colleagues in 2013, 2014, and 2018, respectively, report the usage of a multiepitopic protein for immunization, of which at least one epitope is discontinuous [125,131,132]. This multiepitopic protein protected immunized mice against an intraperitoneal challenge of 2.5 LD_50_s of *L. intermedia* spider venom [125]. The multiepitopic approach proved to generate antiserum with equal neutralization capacity of *L. intermedia* spider venom, as immunization with the recombinant LiD1 toxin alone, except for edema neutralization [131]. Future studies will show if the approach can be utilized to incorporate epitopes from toxins across several spider species and see if they can yield a protective antiserum against the chosen species [125]. This latter approach could, in the future, be attempted in other antivenom fields. Perhaps, by increasing the size of the recombinant protein, immunogenicity of the individual epitopes could be increased. However, a possible obstacle in designing multiepitopic proteins may be ensuring proper epitope folding, and here, linker length might play an essential role [141]. As with the scorpion antivenom field, the spider antivenom development field has yet to explore DNA immunization.

The success of immunizing with recombinant toxins to generate antivenom seems to be determined by the immunogenicity of the immunogen [136], immunization dosages (linked to toxicity), and careful selection of epitopes, which is critical for immunization [136]. Likewise, homology to other toxins in the same venom seems to be important for high neutralization capacity [101]. In order to increase antiserum efficacy and cross-reactivity to closely related toxins from other species, selecting epitopes with high homology to such other toxin components is important [101,109]. However, the utilization of consensus toxins could be a better way to ensure cross-reactivity across homologous toxins, regardless of evolutionary relatedness [75].

## 4. Alternative Venom-Dependent Immunization Approaches

Other than venom-independent strategies, immunization approaches utilizing whole venom, venom-derived toxin fractions, or purified toxins from venom that have been chemically deactivated (toxoids) have also been investigated. Within the venom-dependent approaches, novel delivery systems, adjuvants, and detoxification strategies have been introduced to enhance immune responses and antibody neutralization capacity. In relation to delivery systems, already by 1985, New and colleagues employed sphingomyelin-cholesterol liposomes to encapsulate *Echis carinatus* snake venom, in order to raise an efficacious immune response in mice, rabbits, and sheep [143]. Other studies also utilized liposomes with successful results [77,144,145,146,147,148,149]. For instance, in 1988, Laing et al. encapsulated *E. carinatus* snake venom in sphingomyelin-cholesterol liposomes, and utilized these to immunize mice [144]. Likewise, Freitas et al. reported immunization of mice and rabbits with encapsulated *Crotalus durissus* snake venom, providing better protection than antiserum generated by conventional methods (i.e., using Freund’s complete adjuvant), whilst also using 3 times lower quantity of antigen [145]. In 1991, Chávez-Olórtegui encapsulated *Tityus serrulatus* scorpion venom in sphingomyelin-cholesterol liposomes and used these for immunization of mice, which yielded an antiserum that provided protection in 3 out of 4 naïve mice, when these were challenged with *T. serrulatus* scorpion venom [146]. In 1993, Laing & Theakston demonstrated protection in immunized mice against 4.3 LD_50_s (subcutaneous route) of *E. ocellatus* venom after immunization with venom and lipopolysaccharide (LPS) encapsulated in membrane-stabilized reverse phase evaporation liposomes [147]. Fonseca et al. also reported immunization of mice with encapsulated *T. serrulatus* scorpion venom, providing antiserum with neutralization capacity of up to 3 LD_50_s (subcutaneous route) against Tst-G50 in naïve mice [148].

Other venom-dependent immunization strategies that have been investigated include the utilization of cross-linked chitosan, calcium-alginate, or poly (d,l-lactide) polymer nanoparticles [150,151,152,153]. Cross-linked chitosan nanoparticles were used to encapsulate *T. serrulatus* scorpion venom for immunization of mice, resulting in antigen-specific murine antiserum with IgG titers equal to antisera raised by immunization using an aluminum hydroxide adjuvant [150]. Similar results were achieved by encapsulating *Bothrops jararaca* and *B. erythromelas* snake venom [153], and in another study, calcium-alginate nanoparticles were used to encapsulate *A. australis hector* scorpion venom and applied for immunization of rabbits. The immunized rabbits were protected when challenged subcutaneously with 6 LD_50_s of *A. australis hector* scorpion venom [151]. Lastly, lethality was reported neutralized in naïve mice, when pre-incubating whole venom with murine antiserum from different mice immunized with either *A. australis hector* (258 µg venom neutralized per mL antiserum) or *Buthus occitanus tunetanus* (186 µg venom neutralized per mL antiserum) scorpion venoms and injected by the intracerebroventricular route, where immunogens were encapsulated in poly (d,l-lactide) polymers [152]. Variations in the design of emulsions used as adjuvants have also proven to impact the immune response of animals towards snake venoms [154]. As such, optimization of immunization strategies involves the study of immunological modulation of some venoms affecting the response to other venoms, e.g., when co-immunizing with *B. asper* and *Lachesis stenophrys* snake venoms (see for example [155]). Such knowledge allows for the improved design of immunization protocols, whereby different venoms are injected into animals at different times to avoid such negative modulation.

A third venom-dependent approach that has been investigated has included chemical deactivation of whole venom or toxin fractions used to generate antiserum with an increased neutralization capacity, by enabling higher immunization dosages due to lowered toxicity. This deactivation has been mediated by gamma radiation [156,157,158,159], reaction with glutaraldehyde [160,161,162,163], iodination [164,165], alkylation [166], or reaction with formaldehyde [119]. In 1998, very high neutralization capacities were reported by Heneine & Heneine, who used rabbit antiserum to neutralize up to 30 LD_50_s of *T. serrulatus* scorpion venom in vitro [165]. Likewise, *C. durissus terrificus* snake venom was also iodinated and used for immunization, yielding horse antiserum that protected naïve mice against a challenge of 1.33 mg of *C. durissus terrificus* snake venom. In the same study, *B. jararaca* snake venom was also iodinated and used for immunization of horses, yielding an antivenom that could neutralize 13.7 mg of *B. jararaca* snake venom per mL in naïve mice [165]. These impressive numbers were obtained by abrogating the toxicity of the antigen by gradual iodination, ensuring iodination of tyrosine and histidine residues. The iodination did not oxidize side chains of other amino acids, e.g., indole side chains of tryptophan residues, which may otherwise bring unwanted side effects, such as lowered immunogenicity or antibodies specific against non-native toxins. In this way, Heneine & Heneine obtained detoxified monomeric *T. serrulatus* toxins with assumed conserved epitopes, without experiencing protein aggregation [165].

## 5. Alternative Immunization Approaches from Other Research Fields

In other fields, many new immunization or vaccination approaches have been investigated, which could be used as inspiration for antivenom development, with a few selected examples provided in the following. One approach involves the utilization of extracellular vesicles as a novel delivery system, which can even contain MHC complexes when derived from dendritic cells [167]. It has also been suggested that microvesicles normally carrying nucleic acids could be engineered to carry DNA for immunization [167]. 

Another new delivery approach that has been studied utilizes bacterial ghosts [168,169,170], where a native bacterial outer membrane (with or without LPS [170]) is utilized to encapsulate the desired antigen. This was reported to be effective in inducing dendritic cell maturation in comparison to LPS-based protocols [168], but may carry the risk of a too-low specific antibody titer. A third approach has been to use nanocarriers to encapsulate an antigenic payload [91,171,172,173]. Active nanocarriers are encapsulating nanoparticles with incorporated molecules for targeting purposes. A nanocarrier could for instance incorporate an anti-DC-SIGN/CD209a antibody in the nanocarrier membrane, targeting the vesicle for the mouse DC-SIGN/CD209a receptor on the surface of bone marrow-derived dendritic cells (and macrophages). Schetters et al. reported in 2018 specific targeting of mDC-SIGN^+^ skin dendritic cells upon subcutaneous injection of an OVA-coupled anti-mDC-SIGN antibody, leading to a strong anti-OVA response in vivo [174].

A fourth approach has involved conjugation of the desired antigen to virus-like particles (VLPs), wherein different conjugation techniques have been tested. Fusion proteins and chemical conjugation have been applied for this purpose, but even though much research has gone into this field, few licensed VLP-based vaccines have been marketed [175]. The use of VLP-based vaccines has been extensively reviewed elsewhere [175], but worth highlighting is the bacterial superglue SpyTag/SpyCatcher conjugation method, which has already been used for the development of candidate cancer [176] and malaria vaccines [177,178], among others. In 2014, this technology was used to induce potent B and T cell responses by targeting dendritic cells with specific model antigens. Here, functional synthetic vaccines were assembled by spontaneous conjugation of the targeting vector (i.e., a dendritic cell-targeting antibody fragment fused to SpyTag) and the payload (i.e., an antigen fused to SpyCatcher) [179].

A last approach to be mentioned here is the use of the adenylate cyclase toxin, CyaA, from *Bordetella pertussis*, which mediates translocation of its genetically engineerable N-terminus into the cytoplasmic site of eukaryotic cells with which it is in close proximity [180]. The CyaA toxin has further been engineered to deliver antigens into APCs in vivo, after which epitopes of the antigen have been reported to be confirmed displayed on the MHC I and II complexes of the targeted APCs [180]. In the future, CyaA can be combined with ex vivo dendrite cell vaccinations, where dendrites are cultured ex vivo and re-introduced to the patient [181] (or re-introduced to animals for the production of antivenom), for a more direct targeting approach, but may not be feasible for industrial scale-up in antivenom manufacture.

## 6. Biochemical, Bioinformatic, and Omics Tools that Could Aid Antivenom Development

**Figure 3:**
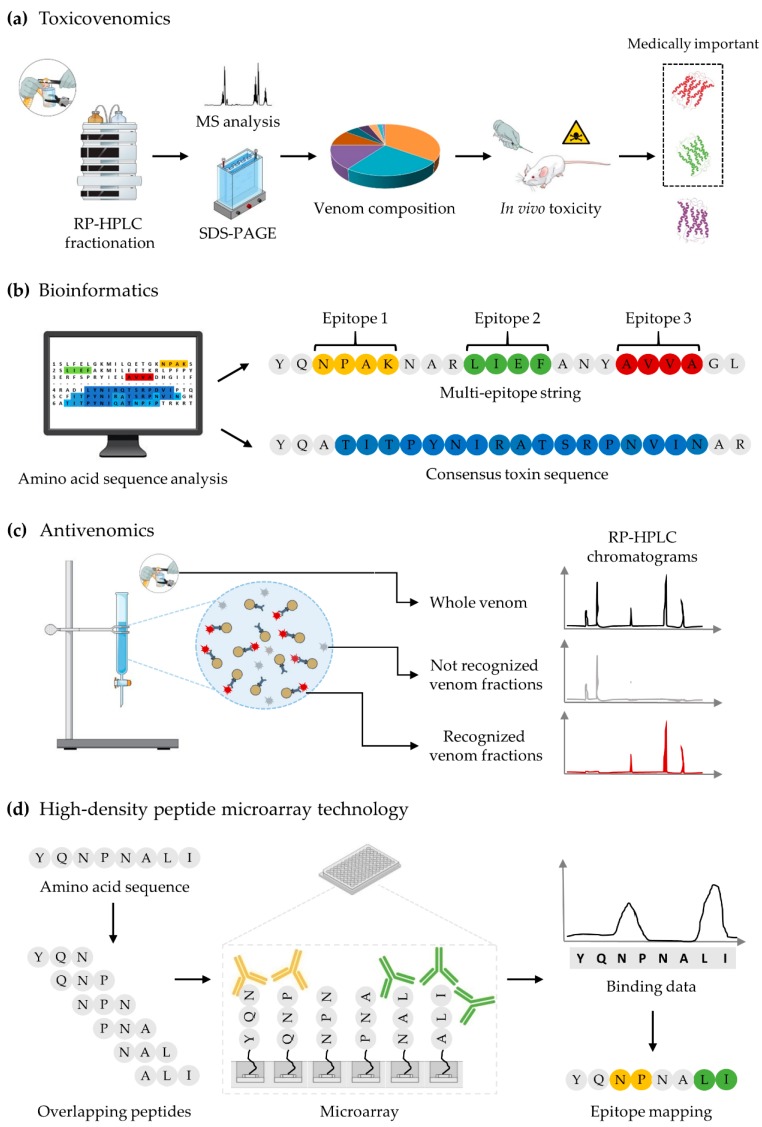
Schematic illustration of biochemical, bioinformatic, and omics tools that could aid antivenom development. (**a**) Toxicovenomics: Venom composition is determined through venomics. Here, venom is fractionated by reversed-phase high-performance liquid chromatography (RP-HPLC). Subsequently, components of each venom fraction are identified by combining sodium dodecyl sulfate-polyacrylamide gel electrophoresis (SDS-PAGE) and mass spectrometry (MS) analysis. The toxicity of each venom fraction is evaluated in vivo to identify the medically relevant components an antivenom should target. (**b**) Bioinformatics: Online resources can be used for in silico prediction of epitopic elements and regions of homology from toxin sequences. Predicted epitopes and homologous regions can then be utilized to generate multi-epitopic strings or consensus toxin sequences, respectively. Such strategies aim at generating an immune response against multiple toxins using a single molecule or to generate cross-reactive antibodies. (**c**) Antivenomics: This approach is employed to assess antivenom reactivity and cross-reactivity based on immunoaffinity. By comparing the chromatographic profiles of whole venom, unbound, and bound venom components, it is possible to discriminate between venom fractions recognized and not recognized by antivenom antibodies. (**d**) High-density peptide microarray technology: Short overlapping peptides from toxin amino acid sequences are synthesized to study epitope-paratope interactions at an amino acid level by incubating the peptides with antivenom antibodies. This strategy can be used to map epitopes or to assess antivenom reactivity and cross-reactivity in a high-throughput manner.

Due to the complex composition of snake, scorpion, and spider venoms [33,182], the development of next-generation antivenoms is not a trivial matter. Regardless of the developmental strategy explored, it has become a necessity to establish which toxins researchers should focus on to achieve neutralization of venom toxicity [42,183]. To achieve this, biochemical, bioinformatic, and omics tools can be utilized to assist the process of antivenom development. The determination of key toxin targets involves a two-step process. First, a detailed overview of venom composition(s) is required. For this purpose, venom proteomics analysis (i.e., venomics) can be performed. Venomics allows for the identification and quantification of venom components by combining venom fractionation using high performance liquid chromatography and gel electrophoresis with mass spectrometry [40,47,184,185,186,187]. A more comprehensive venom profile can be obtained by integrating proteomics with genomic and transcriptomic data [30,188], although mRNA transcription and protein expression levels do not always correlate well [189]. Second, venomics data should be complemented with in vivo toxicity studies of each toxin or venom fraction (i.e., toxicovenomics) to determine which components are crucial for toxicity (Figure 3a) [190,191,192]. Using both venomics and toxicovenomics data, it is possible to select specific key toxins based on their relative medical importance [42], and exclusively use these to immunize animals.

In search for key toxin targets, nowadays, numerous web servers for in silico prediction of continuous and/or discontinuous epitopic elements have been developed (reviewed in [193,194,195,196,197,198,199,200]). Based on either amino acid sequence analysis [201,202,203,204,205,206,207,208] or 3D-structural data [205,209,210,211,212], these bioinformatic resources circumvent the expensive and time-consuming experimental procedures previously employed for epitope mapping (Figure 3b). Localizing the immunogenic region of toxins may allow for the synthesis of small peptides that could be used as synthetic epitopes for immunization. Generally, neutralizing antibodies target epitopes that are three-dimensional structures that may contain discontinuous epitopic elements [213]. However, some continuous epitopic elements are still able to raise a protective immune response, as extensively exemplified by the studies reported in Table 1, Table 2 and Table 3. In addition, shortening the size of the required antigen has opened the possibility of generating recombinant epitope strings, in which immunization with a single molecule can give rise to an immune response against multiple toxins [66,72,125]. Bioinformatic tools have also been applied for identification of regions of homology between toxins belonging to the same family to determine consensus toxin sequences [75,83]. Immunization with proteins derived from consensus sequences aims towards generating cross-reactive antibodies (i.e., antibodies with the ability to recognize multiple structurally similar toxins), ideally with cross-neutralization potential.

Cross-neutralizing antibodies are of utmost value, since they could be used to formulate polyvalent antivenoms [30]. Besides the classical ELISA and enzymatic neutralization assays employed for in vitro assessment of antivenom reactivity and cross-reactivity, more recently, antivenomics has been employed for this purpose (Figure 3c). Basically, whole venom is passed through a chromatographic column to which antivenom antibodies have been immobilized to. Due to immunoaffinity, venom components recognized by antivenom antibodies are captured. Therefore, by comparing the chromatographic profiles of whole venom, unbound, and bound venom components, antivenomics can be used to determine which toxins the antivenom has the capacity to bind [26,40,214,215,216,217]. Despite providing a detailed overview of the antivenom’s reactivity against individual venom toxins, this strategy does not give precise information about the epitopic elements involved in toxin-antibody interactions. In order to increase the resolution of epitope-paratope interactions, high-density peptide microarray technology has recently been implemented in antivenom research (Figure 3d). With this technology, the immunoreactivity of antivenoms against numerous continuous epitopic elements derived from toxin sequences can be evaluated at an amino acid level in a high-throughput manner [218,219,220]. Although cross-neutralization strictly needs to be assessed in in vivo studies, antivenomics and high-density peptide microarray technology allow for comprehensive analysis of antivenom cross-reactivity in a rapid and relatively inexpensive way [30]. These tools may thus provide important guidance for directing the development of novel antivenoms [30].

## 7. Conclusions

Improving the efficacy, safety, and affordability of antivenoms against animal envenomings should be a key priority to enable better treatment of these diseases that particularly affect impoverished victims in the rural tropics [12,221,222,223,224]. In the far future, a possibility may exist for introducing novel antivenom products based on recombinantly produced antibodies or small molecule inhibitors [35,36,225,226]. However, immediate improvements are also warranted, where innovative, venom-independent immunization approaches offer the potential of quick adaptability, as they are compatible with existing antivenom manufacturing setups. In addition, by circumventing the need for venom in antivenom manufacture, the complications associated with animal captivity and venom collection are to some extent limited.

Antivenom development by means of novel immunization approaches has been possible due to the growing knowledge on venom compositions and toxicity obtained through different omics technologies and other biotechnological tools, such as epitope mapping and recombinant expression of heterologous proteins. Such approaches could particularly be used to improve efficacy (higher therapeutic antibody titers) [33] and to broaden the neutralization capacity of antivenoms to cover species for which none of the currently available antivenoms work [227]. Improving these properties may in turn lead to improved affordability for envenomed victims, as fewer antivenom vials with a higher content of therapeutic antibodies would be needed to treat an envenoming. Moreover, broadening the neutralization capacity of antivenoms could possibly warrant larger manufacturing outputs, thereby decreasing cost of manufacture per vial. However, despite the numerous accounts of the successful use of novel immunization strategies reported by different antivenom research laboratories, very few of these strategies have found their way to the industrial setting. In the future, the possibility also exists that a combination of innovative immunization strategies with transgenic animals expressing the human antibody repertoire [228,229,230,231] could be employed in antivenom manufacture. This may not improve efficacy or affordability, however, it may indeed provide safer antivenoms, as the therapeutic antibodies would be of human, rather than animal origin. Improving safety may even allow for earlier administration of antivenom to victims, which could possibly improve treatment and recovery, due to a lowered risk of adverse reactions.

## Figures and Tables

**Table 1:** Reported work on innovative venom-independent immunization strategies for snake antivenom development.

Authors & Year	Immunization Strategy	Species	Target Toxin(s)	Antivenom	Challenge Toxin(s)	Effect(s) Neutralized	Ref.
Čurin-Šerbec et al., 1991	Synthetic epitope	*Vipera ammodytes*	Ammodytoxin A	Rabbit antiserum	Ammodytoxin A	Lethality	[70]
Čurin-Šerbec et al., 1994	Synthetic epitope	*Crotalus durissus terrificus*, *Vipera ammodytes*	Crotoxin and ammodytoxin A	Murine IgG, IgM	Crotoxin	Prolonged survival time	[68]
Dolimbek and Atassi, 1996	Synthetic epitope	*Bungarus multicinctus*	α-bungarotoxin	Murine antiserum	α-bungarotoxin	Lethality	[71]
Calderón et al., 1999	Synthetic epitope	*Bothrops asper*	Myotoxin II	Murine antiserum	Myotoxin II	Myotoxicity	[67]
Harrison et al., 2000	DNA	*Bothrops jararaca*	Jararhagin	Murine antiserum	*B. jararaca* venom	Myotoxicity	[79]
Harrison et al., 2002	DNA	*Bothrops jararaca*	Jararhagin	Murine antiserum	N/A	Not Evaluated	[80]
Pergolizzi et al., 2004	DNA	*Naja kaouthia*	α-cobratoxin	Murine antiserum	α-cobratoxin	Lethality	[85]
Wagstaff et al., 2006	DNA	*Echis ocellatus*	SVMPs	Murine IgG	*E. ocellatus* and *Cerastes cerastes* venom	Hemotoxicity	[83]
Ferreira et al., 2006	Synthetic epitope	*Lachesis muta muta*	Mutalysin II	Rabbit IgG	Mutalysin II	Hemotoxicity	[69]
Azofeifa-Cordero et al., 2008	DNA	*Crotalus durissus durissus*	P-III SVMP	Murine antiserum	*C. d. durissus* venom	Hemotoxicity	[84]
Leão et al., 2009	DNA	*Micrurus corallinus*	3FTx and PLA2s	Murine antiserum	N/A	Not evaluated	[95]
Cardoso et al., 2009	Recombinant mimotope	*Bothrops neuwiedi*	Neuwiedase	Murine antiserum	N/A	Not evaluated	[78]
Arce-Estrada et al., 2009	DNA	*Bothrops asper*	P-II SVMP	Equine antiserum	*B. asper* and *C. d. durissus* venom	Hemotoxicity	[82]
Suntrarachun et al., 2010	Recombinant toxin	*Bungarus candidus*	α and β-neurotoxins	Murine antiserum	*B. candidus* venom	Lethality	[73]
Machado de Avila et al., 2011	Synthetic mimotope	*Lachesis muta*	Mutalysin II	Rabbit antiserum	*L. muta* venom	Hemotoxicity	[77]
Ramos et al., 2016	DNA + Recombinant protein	*Micrurus corallinus*	3FTxs and PLA_2_	Murine antiserum	*M. corallinus* venom	Lethality	[66]
Cao et al., 2016	Recombinant protein	*Deinagkistrodon acutus*	SVSPs, SVMPs and PLA_2_s	Murine antiserum	*D. acutus* venom	Hemotoxicity	[72]
Clement et al., 2016	Recombinant toxin	*Micrurus laticorallis*	Cysteine-rich neurotoxins (Mlat1)	Rabbit antiserum	*M. laticorallis* venom	Not neutralizing	[96]
Hasson, 2017	DNA	*Echis ocellatus*	Disintegrin	Murine antiserum	*Crotalus atrox, E. ocellatus and Bitis arietans* venom	Hemotoxicity	[81]
de la Rosa et al., 2018	Recombinant toxin	*Acanthophis* spp., *Oxyuranus* spp., *Walterinnesia* spp., *Naja* spp., *Dendroaspis* spp. and *Micrurus* spp.	Type I α-neurotoxins	Rabbit antiserum	N/A	Not evaluated	[75]
Guerrero-Garzón et al., 2018	Recombinant toxin	*Micrurus diastema*	Type I α-neurotoxin D.H.	Rabbit antiserum	rD.H, MlatA1, and fraction F5 from *M. diastema* venom	Lethality	[74]

**Table 2:** Reported work on innovative venom-independent immunization strategies for scorpion antivenom development.

Authors & Year	Immunization Strategy	Species	Target Toxin(s)	Antivenom	Challenge Toxin(s)	Effect(s) Neutralized	Ref.
Bahraoui et al., 1986	Synthetic epitope	*Androctonus australis hector*	Toxin II (AahII)	Murine antiserum	AahII	Lethality	[97]
Devaux et al., 1993	Synthetic epitope	*Androctonus australis hector*	Toxin II (AahII)	Rabbit antiserum	N/A	N/A	[103]
Calderón-Aranda et al., 1995	Synthetic epitope	*Centruroides noxius*	Cn2	Rabbit and murine antisera	Cn2	Lethality	[98]
Bouhaouala-Zahar et al., 1996	Recombinant toxin	*Buthus occitanus tunetanus*	α-toxin	Murine antiserum	Bot and AaHG	Lethality	[121]
Devaux et al., 1997	Synthetic peptide	*Androctonus australis hector*	Toxin II (AahII)	Murine IgG	AahII	Lethality	[108]
Zenouaki et al., 1997	Synthetic peptide	*Androctonus australis hector*	Toxin II (AahII)	Rabbit antiserum	AahII	Lethality	[106]
Calderón-Aranda et al., 1999	Synthetic epitope	*Centruroides noxius*	Cn2	Rabbit and murine antisera	Cn2	Lethality	[99]
Guatimosim et al., 2000	Recombinant toxoid	*Tityus serrulatus*	TsNTxP	Rabbit antiserum	*T. serrulatus* venom	Lethality	[109]
Gazarian et al., 2000	Mimotopes	*Centruroides noxius* Hoffmann	Noxiustoxin	Murine antiserum	N/A	N/A	[122]
Chávez-Olórtegui et al., 2001	Synthetic epitope	*Tityus serrulatus*	TsNTxP	Rabbit antiserum	TstG50	Lethality	[104]
Legros et al., 2001	Recombinant toxin	*Androctonus australis hector*	AahI, AahII and AahIII (α-toxins)	Rabbit and murine antisera	AaH-G50	Lethality	[110]
Benkhadir et al., 2002	Recombinant toxin	*Buthus occitanus tunetanus*	Bot III (α-toxin)	Murine antiserum	*B. occitanus tunetanus* venom	Lethality	[111]
Alvarenga et al., 2002	Synthetic epitope	*Tityus serrulatus*	TsNTxP and TsIV	Rabbit antiserum	TstG50	Lethality	[100]
Garcia et al., 2003	Recombinant toxin	*Centruroides noxius* Hoffmann	Cn5 and sub-fraction	Rabbit antiserum	Cn2	Lethality	[112]
Inceoglu et al., 2006	Synthetic epitope	*Parabuthus transvaalicus*	Birtoxin	Rabbit polyclonal IgG	*P. transvaalicus* venom	Lethality	[101]
Corona Villegas et al., 2008	Recombinant toxin	*Centruroides* spp.	Cex1-13, Cll3-8, Cn4b, Cn10b, Ce3, Ce5-7, Ce13(b), Cg1-3, CsEv1-3, CsEV8-9, CsE1x, CsEIa, CexErg1-4, Cll Erg1-4, Cn Erg3-5, CeErg1-3, CgErg1-3, CsErg1-5	Rabbit antiserum	Cn2	Lethality	[113]
Mendes et al., 2008	Recombinant toxin	*Tityus serrulatus*	Ts1	Rabbit antiserum	Tst1 and *T. serrulatus* venom	Lethality	[114]
Hernández-Salgado et al., 2009	Recombinant toxin and toxoid	*Centruroides suffusus suffusus*	CssII	Rabbit antiserum	CssII, Cn2, and *C. suffusus suffusus* venom	Lethality	[115]
García-Gómez et al., 2009	Recombinant toxin	*Parabuthus granulatus*	Pg8	Murine antiserum	Pg8 and *P. granulatus* venom	Lethality	[116]
Duarte et al., 2010	Synthetic epitope	*Tityus serrulatus*	TsNTxP	Murine antiserum	*T. serrulatus* venom	Lethality	[102]
Eskandari et al., 2014	Recombinant toxin	*Mesobuthus eupeus*	BMK neurotoxin	Murine antiserum	N/A	Not evaluated	[117]
Uawonggul et al., 2014	Recombinant toxin	*Heterometrus laoticus*	Heteroscorpine-1 (HS-1)	Murine antiserum	*H. laoticus* venom	Paralysis	[120]
Jiménez-Vargas et al., 2017	Recombinant toxin	*Centruroides* spp.	Cn2, Css2, Cll1, and Cll2	Murine and rabbit antisera	*C. noxius*, *C. suffusus*, *C. limpidus*, *C. elegans*, *C. tecomanus*, and *C. sculpturatus* venom	Lethality	[118]
Safari Foroushani et al., 2018	Recombinant toxoid	*Hemiscorpius lepturus*	rPLD1	Murine antiserum	rPLD1 and *H. lepturus* venom	Lethality	[119]

**Table 3:** Reported work on innovative venom-independent immunization strategies for spider antivenom development.

Authors & Year	Immunization Strategy	Species	Target Toxin(s)	Antivenom	Challenge Toxin(s)	Effect(s) Neutralized	Ref.
Fernandes Pedrosa et al., 2002	Recombinant toxin	*Loxosceles laeta*	Smase I	Rabbit antiserum	rSmase I and *L. laeta* venom	Dermonecrosis	[139]
Araujo et al., 2003	Recombinant toxin	*Loxosceles intermedia*	Dermonecrotic toxin	Murine antiserum	*L. intermedia* venom	Dermonecrosis and lethality	[127]
Tambourgi et al., 2004	Recombinant toxin	*Loxosceles intermedia*	Sphingomyelinases	Rabbit antiserum	N/A	N/A	[123]
Olvera et al., 2006	Recombinant toxin	*Loxosceles reclusa*, *Loxosceles boneti*, *Loxosceles laeta*	Sphingomyelinase D	Rabbit antiserum and equine F(ab’)_2_	rSMD, *L. reclusa*, *L. boneti* and *L. laeta* venom	Lethality	[128]
Felicori et al., 2006	Recombinant toxin	*Loxosceles intermedia*	Dermonecrotic toxin LiD1	Murine antiserum	*L. intermedia* venom	Lethality	[129]
Fischer et al., 2007	Synthetic epitope	*Atrax robustus*	Robustoxin	Murine antiserum	*A. robustus* venom	Lethality	[107]
de Almeida et al., 2008	Recombinant toxin	*Loxosceles intermedia, Loxosceles laeta* and *Loxosceles gaucho*	Sphingomyelinase D	Equine antiserum	*L. intermedia*, *L. laeta*, and *L. gaucho* venom	Dermonecrosis	[130]
Felicori et al., 2009	Synthetic epitope	*Loxosceles intermedia*	Dermonecrotic toxin LiD1	Rabbit IgGs	LiD1	Dermonecrosis, hemotoxicity, and edema	[136]
Comis et al., 2009	Synthetic toxin	*Atrax robustus*	Robustoxin	Monkey antiserum	*A. robustus* venom	Lethality	[126]
Dias-Lopes et al., 2010	Synthetic epitope	*Loxosceles intermedia*	Dermonecrotic toxin LiD1	Rabbit and murine antisera	rLiD1 and *L. intermedia* venom	Dermonecrosis, hemotoxicity, and lethality	[137]
Chaim et al., 2011	Recombinant toxoid	*Loxosceles intermedia*	Dermonecrotic toxin LiD1	Rabbit antiserum	N/A	N/A	[142]
de Moura et al., 2011	Synthetic mimotope	*Loxosceles intermedia*	Dermonecrotic toxin LiD1	Rabbit antiserum	*L. intermedia* venom	Dermonecrosis, hemotoxicity	[138]
Mendes et al., 2013	Recombinant toxin	*Loxosceles intermedia*	Dermonecrotic toxin LiD1	Rabbit antiserum and IgG	rLiD1	Dermonecrosis, hemotoxicity	[131]
Magalhães et al., 2013	Recombinant toxin	*Loxosceles gaucho*	Phospholipase D	Rabbit antiserum	LgRec1 and *L. gaucho* venom	Dermonecrosis, local reaction	[124]
Figueiredo et al., 2014	Recombinant toxin	*Loxosceles intermedia, Loxosceles laeta*, and *Loxosceles gaucho*	Sphingomyelinase D	Equine antiserum	*L. intermedia, L. gaucho* and *L. laeta* venom	Dermonecrosis	[132]
Dias-Lopes et al., 2014	Recombinant toxin	*Loxosceles intermedia*, *Loxosceles laeta*, and *Loxosceles gaucho*	Sphingomyelinase D	Murine IgG	rLiD1	Dermonecrosis, hemotoxicity, and edema	[133]
Duarte et al., 2015	Recombinant toxin	*Loxosceles intermedia* and *Loxosceles laeta*	Dermonecrotic toxin LiD1	Equine antiserum	*L. intermedia* and *L. laeta* venom	Dermonecrosis, hemotoxicity	[134]
Oliveira et al., 2016	Recombinant toxin	*Loxosceles intermedia*	Sphingomyelinase D (SMD)	Murine antiserum	rSMDs, *L. reclusa*, *L. boneti*, and *L. laeta* venom	Lethality	[135]
Lima et al., 2018	Recombinant toxin	*Loxosceles intermedia* and *Loxosceles laeta*	Loxosceles astacin-like protease 1, hyaluronidases, SMase-I	Rabbit antiserum	*L. intermedia* venom	Lethality	[125]
